# Sinapic Acid Attenuates LPS‐Induced Acute Kidney Injury in Rats: Changes in Autophagy‐Related, Apoptotic, Inflammatory, and Oxidative Stress Markers

**DOI:** 10.1002/jbt.71075

**Published:** 2026-08-12

**Authors:** Serdar Doğan, Hamza Malik Okuyan, Ayça Coşkun, Şeyda Öznur Ayçiçek Özen, Mehmet Doğan, İhsan Karaboğa, Sibel Kulaksızoğlu

**Affiliations:** ^1^ Department of Biochemistry Antalya City Hospital Antalya Türkiye; ^2^ Department of Physiotherapy and Rehabilitation ‐ Faculty of Health Sciences, Biomedical Technologies Application and Research Center Sakarya University of Applied Sciences Sakarya Türkiye; ^3^ Department of Molecular Biology and Genetics, Faculty of Science Kilis 7 Aralık University Kilis Türkiye; ^4^ Department of Histology and Embryology, Faculty of Medicine Kırklareli University Kırklareli Türkiye; ^5^ Medical Biochemistry Laboratory Antalya Training and Research Hospital Antalya Türkiye

**Keywords:** acute kidney injury, apoptosis, autophagy, inflammation, LPS, oxidative stress, sinapic acid

## Abstract

Lipopolysaccharide (LPS)‐induced acute kidney injury (AKI) is associated with high morbidity and mortality. The molecular mechanisms underlying sepsis‐associated renal injury remain incompletely understood. Sinapic acid (SA), a bioactive phenolic compound, exhibits antioxidant, anti‐inflammatory, and cytoprotective properties, but its nephroprotective role in LPS‐induced AKI has not been clarified. We evaluated the protective effects of SA in an LPS‐induced AKI rat model and examined its associations with autophagy‐related, apoptotic, inflammatory, and oxidative stress markers. AKI was induced by a single intraperitoneal injection of LPS (5 mg/kg) following 7 days of oral SA pretreatment (40 mg/kg/day). LPS administration caused marked renal tubular injury and significantly increased serum BUN, CREA, and UA levels. SA pretreatment significantly attenuated these alterations. Moreover, LPS increased renal BECN1 immunoreactivity and circulating SQSTM1/p62 levels, indicating alterations in autophagy‐related markers, together with increased renal TNF‐α and Caspase‐3 immunoreactivity. SA pretreatment significantly attenuated the LPS‐induced increases in these markers. LPS also increased renal MDA levels and serum total oxidant status. SA pretreatment significantly reduced renal MDA without significantly altering SOD, GPx, or total antioxidant status, indicating attenuation of lipid peroxidation rather than a generalized enhancement of antioxidant defenses. Furthermore, SA attenuated the LPS‐induced reductions in serum albumin and total protein and the increase in LDH. Collectively, these findings suggest that SA pretreatment attenuates LPS‐induced AKI and that this protective effect is accompanied by changes in autophagy‐related markers, reduced renal TNF‐α and Caspase‐3 immunoreactivity, and decreased renal lipid peroxidation.

## Summary

- SA pretreatment attenuates LPS‐induced renal tubular injury.
- SA improves serum markers of renal dysfunction following LPS exposure.
- SA attenuates changes in the autophagy‐related markers BECN1 and SQSTM1.
- SA reduces renal TNF‐α and Caspase‐3 immunoreactivity.
- SA reduces renal lipid peroxidation without enhancing antioxidant defenses.

## Graphical abstract

Schematic representation of the effects of SA pretreatment in rats with LPS‐induced AKI. LPS administration increased renal BECN1, TNF‐α, and Caspase‐3 immunoreactivity, circulating SQSTM1/p62 levels, and renal MDA levels. These changes were accompanied by renal tubular injury, increased serum BUN, CREA, UA, and LDH levels, and reduced ALB and TP levels. SA pretreatment attenuated renal tubular injury and these LPS‐induced biochemical and marker‐level alterations. Overall, the protective effects of SA were accompanied by changes in autophagy‐related markers and reductions in renal TNF‐α and Caspase‐3 immunoreactivity and lipid peroxidation. AKI, acute kidney injury; ALB, albumin; BECN1, Beclin‐1; BUN, blood urea nitrogen; Caspase‐3, cysteine‐aspartic protease‐3; CREA, creatinine; LDH, lactate dehydrogenase; LPS, lipopolysaccharide; MDA, malondialdehyde; SA, sinapic acid; SQSTM1, sequestosome‐1; TNF‐α, tumor necrosis factor‐alpha; TP, total protein; UA, uric acid. (Created in BioRender. Okuyan, H. M. (2026) https://BioRender.com/2cd16tu)

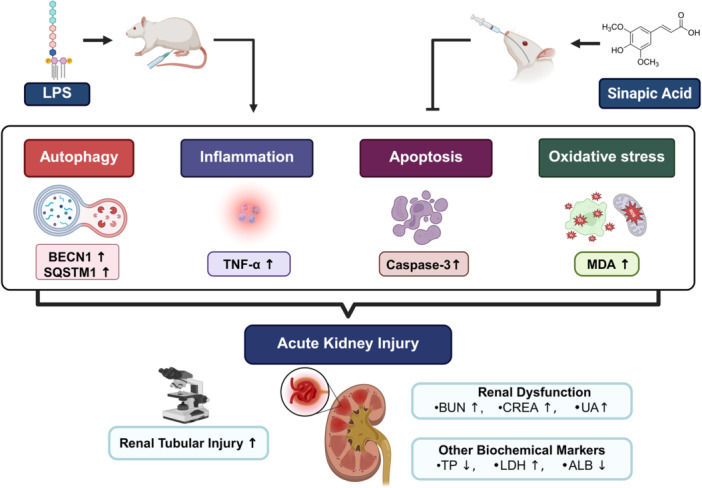

## 1 Introduction

Sepsis represents a multifaceted clinical syndrome characterized by an aberrant host response to infectious agents, culminating in potentially fatal organ dysfunction. Sepsis‐induced acute kidney injury (SI‐AKI) is one of its most severe complications and a major contributor to morbidity and mortality in critically sick patients [1]. Lipopolysaccharide (LPS) endotoxin–induced sepsis is a common cause of AKI and a potent driver of inflammatory signaling pathways, resulting in the dysfunction of multiple organ systems. The LPS‐induced AKI model is extensively employed to investigate renal dysfunction and the underlying pathophysiological mechanisms [2]. The mechanisms underlying SI‐AKI are largely driven by aberrant inflammatory responses, impaired autophagy, oxidative stress, and endoplasmic reticulum stress, which collectively disrupt cellular homeostasis and aggravate renal tissue injury [2]. Autophagy is a fundamental cellular process essential for maintaining physiological homeostasis. It plays a significant role in the pathophysiology of LPS‐induced AKI, affecting cell viability, apoptosis, and inflammatory responses. The modulation of autophagy may present a promising therapeutic avenue for the management of AKI [3]. Beclin‐1 (BECN1), an essential modulator protein of the autophagic process, exhibits increased expression in renal tissue during LPS‐induced AKI. Depending on the specific context and the conditions of activation, BECN1‐mediated autophagy may confer either protective or harmful consequences on renal injury [4]. However, Beclin‐1 primarily reflects autophagy initiation and does not, by itself, indicate autophagic flux. SQSTM1/p62, a selective autophagy substrate degraded upon successful autophagosome–lysosome fusion, provides complementary information on autophagic clearance capacity and is widely used alongside initiation markers to interpret the functional status of the autophagic pathway [5]. Tumor necrosis factor‐alpha (TNF‐α) is a crucial pro‐inflammatory cytokine that induces kidney inflammation and damage after exposure to LPS endotoxin. While TNF‐α plays a role in the progression of kidney injury, it is also crucial for the initiation of preventive immune responses. Consequently, treatment approaches directed at TNF‐α should strive for balanced control to maintain advantageous immune functions while mitigating renal damage [6]. Oxidative stress constitutes a fundamental pathological feature of AKI induced by LPS and plays a significant role in exacerbating renal impairment and inflammatory responses [7]. Exposure to LPS elevates reactive oxygen species (ROS) and malondialdehyde (MDA) levels while concurrently diminishing antioxidant capacity, all of which signify oxidative stress and correlate significantly with the progression and severity of renal injury [8]. In addition to autophagy, inflammation, and oxidative stress, apoptotic tubular epithelial cell death constitutes a further critical mechanism driving LPS‐induced renal injury, with Caspase‐3 activation serving as a key executioner event that links upstream inflammatory and oxidative insults to irreversible cell loss [9]. These findings highlight the necessity to explore therapeutic options that might regulate oxidative stress, inflammation, and autophagy to mitigate renal impairment in LPS‐induced AKI.

Sinapic acid (SA) is a naturally occurring hydroxycinnamic acid recognized for its wide pharmacological activities, owing to its notable antioxidant, anti‐inflammatory, and cytoprotective effects [10]. SA reduces oxidative stress by neutralizing ROS, reducing lipid peroxidation, and increasing natural antioxidant systems such as SOD, CAT, and GPx [11, 12]. The anti‐inflammatory effects of SA are achieved by downregulating pro‐inflammatory cytokines (e.g., TNF‐α, IL‐6, IL‐1β) and inhibiting NF‐κB signaling [13]. Additionally, SA provides renoprotective, hepatoprotective, cardioprotective, neuroprotective, and anti‐diabetic benefits, mostly because to its dual antioxidant and anti‐inflammatory mechanisms [10, 14]. SA has been shown to ameliorate cisplatin‐induced AKI via regulation of apoptosis and oxidative stress; however, its impact on autophagy, inflammation and oxidative stress in LPS‐induced AKI remains unexplored. Considering autophagy, inflammation, and oxidative stress are critical therapeutic targets in LPS‐induced AKI [2, 4, 6], we aimed to determine whether SA pretreatment attenuates LPS‐induced renal injury and whether its protective effects are accompanied by changes in autophagy‐related, apoptosis‐associated, inflammatory, and oxidative stress markers.

## 2 Materials and Methods

### 2.1 Experimental Design

**Figure 1:**
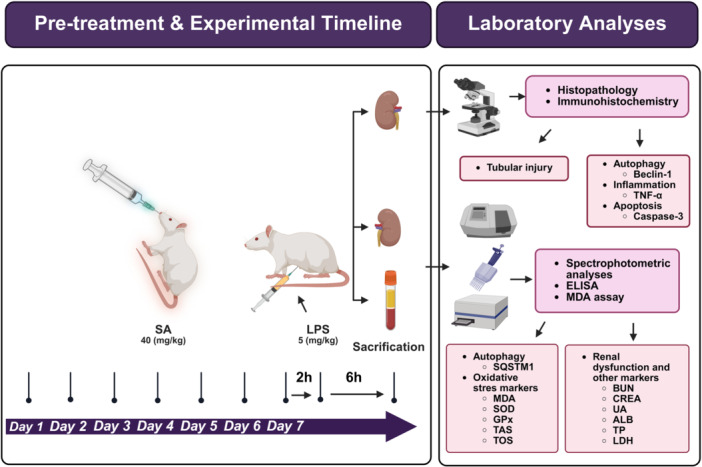
Experimental design and timeline for evaluating the protective effects of SA against LPS‐induced AKI in rats. Rats were orally pretreated with SA (40 mg/kg/day) for 7 consecutive days. On Day 7, LPS (5 mg/kg) was administered intraperitoneally to induce AKI. On Day 7, 2 h after the final SA dose, LPS was administreted intraperitoneally. Rats were monitored, and at 6 h post‐injection, all animals were sacrificed for tissue and blood collection. Histopathological evaluation of kidney tissues was performed to assess tubular injury scores. Immunohistochemistry was conducted to detect Beclin‐1 (autophagy marker), TNF‐α (inflammation marker), and Caspase‐3 (apoptosis marker). Blood samples were analyzed for biochemical markers, including CREA, ALB, TP, BUN, UA, and SQSTM1/p62 (autophagy‐related marker). Oxidative stress parameters, including MDA, SOD, GPx, TAS, and TOS, were quantified in kidney tissues to evaluate the redox balance. AKI, acute kidney injury; ALB, albumin; BECN1, Beclin‐1; BUN, blood urea nitrogen; CREA, creatinine; GPx, glutathione peroxidase; LDH, lactate dehydrogenase; LPS, lipopolysaccharide; MDA, malondialdehyde; SA, Sinapic acid; SOD, superoxide dismutase; SQSTM1, sequestosome 1; TAS, total antioxidant status; TNF‐α, tumor necrosis factor‐alpha; TOS, total oxidant status; TP, total protein; UA, uric acid. Created in BioRender. Okuyan, H. M. (2026) https://BioRender.com/ggmmyv2.

This work adhered to the rules established by the Institutional Animal Care and Use Committee, with all experimental techniques sanctioned by the Local Animal Ethics Committee of Sakarya University (Approval number: 2023‐14). Subsequent to obtaining ethical approval, the animal studies were conducted at Sakarya University's Experimental Medicine Application and Research Center. The research encompassed 24 male *Wistar albino* rats, each weighing between 200 and 250 grams. All animals were granted full access to food and water during the research. We fed the rats a standard commercial diet consisting of 65% carbohydrates, 24% protein, and 11% fat, with a defined metabolizable energy value (Altromin 1324, Lage, Germany). All of the lab animals were kept in a controlled setting with a 12‐h light/dark cycle at a temperature of 21 ± 2°C. We habituated the rats to the laboratory setting for 1 week prior to commencing the research. All rats were randomly allocated into three groups, each comprising eight subjects: (1) Control group, (2) LPS group and (3) LPS + SA group (40 mg/kg). Lipopolysaccharide (LPS; Cat. No. L2630) and Sinapic Acid (SA; Cat. No. D7927) were purchased from Sigma‐Aldrich (USA). As previously described, we established a moderate AKI model in rats by administering a single intraperitoneal dose of LPS (5 mg/kg) [2, 15]. 1. Control (sham) group: Over a period of seven consecutive days, we delivered an equivalent volume of orogastric SA solvent to the rats in this group as was provided to the LPS + SA groups. Furthermore, on the day of LPS administration in the other groups, these rats were administered an equivalent volume of sterile saline. 2. LPS group: We orally delivered an equivalent volume of SA solvent to the rats in this group as provided to the LPS + SA groups for a duration of seven consecutive days. On the seventh day, the rats were administered a single intraperitoneal injection of LPS at a dosage of 5 mg/kg. 3. LPS + SA group: We delivered 40 mg/kg of SA orally to the rats in this group once daily for seven consecutive days. On the seventh day, 2 h after the final SA administration, the rats received a single intraperitoneal injection of LPS (5 mg/kg). 6 h after LPS administration, all rats were anesthetized with ketamine/xylazine (90/10 mg/kg), and blood and kidney samples were collected. We performed histological and immunohistochemical examinations on the right kidney tissues, while we conducted biochemical evaluations on the left kidney tissues. Figure 1 provides a detailed summary of the study design.

### 2.2 Histological Analyzes of Kidney Tissues

The structural assessment of the right kidney tissues was conducted utilizing standard histological techniques and Hematoxylin‐Eosin (H&E) staining. The tissues were fixed in 4% neutral formalin at ambient temperature for 48 h. After fixation, specimens were rinsed overnight in running tap water, then dehydrated using a graded ethanol series (70%, 80%, 90%, 96%, and 100%) and later cleaned with xylene. Tissues embedded in soft and hard paraffin were sectioned after being blocked and processed. The embedding procedure was executed via a paraffin dispenser (Leica HistoCore Arcadia). Sections derived from the tissue blocks were utilized for histological analysis and immunohistochemical staining for BECN1, TNF‐α, and Caspase‐3. A semi‐motorized microtome (Leica RM2245) was used to slice the paraffin‐embedded tissue blocks into 5 µm thick sections. We positioned the resultant slices onto adhesive slides and conducted Hematoxylin‐Eosin (H&E) staining. Subsequently, we acquired photographs of the stained sections utilizing an Olympus BX41 research microscope outfitted with a camera. The tubular damage in kidney tissue was assessed using previously established methodologies [16]. Accordingly; 0, no damage; 1, damaged area 1%–10%; 2, damaged area 11%–25%; 3, damaged area 26%–75%; and 4, damaged area 75% or more [16].

### 2.3 Immunohistochemical Analyzes of BECN1, TNF‐α, and Caspase‐3 in Kidney Tissues

We assessed BECN1, TNF‐α, and Caspase‐3 immunoreactivity in right kidney tissues using an indirect immunohistochemical method. BECN1 was evaluated as an autophagy‐associated protein, TNF‐α as a pro‐inflammatory cytokine, and Caspase‐3 as an apoptosis‐associated protein. The sections were deparaffinized, rehydrated through a graded ethanol series, and immersed in distilled water. After rinsing with phosphate‐buffered saline (PBS), the tissue sections were delineated using a hydrophobic PAP pen. Antigen retrieval was performed by heating the sections in citrate buffer using a microwave oven. Endogenous peroxidase activity was blocked using 3% hydrogen peroxide. After incubation with blocking serum for 1 h at room temperature, the sections were incubated with primary antibodies against BECN1 (1:50, AF5128; Affinity Biosciences), TNF‐α (1:100, AF7014; Affinity Biosciences), and Caspase‐3 (1:50, AF7022; Affinity Biosciences). Subsequently, biotinylated secondary antibodies and streptavidin were applied for 30 min. AEC (3‐amino‐9‐ethylcarbazole) was used as the chromogen, and Mayer's hematoxylin was used for counterstaining. BECN1, TNF‐α, and Caspase‐3 immunoreactivity was semiquantitatively evaluated using the method described by Carter et al. [17]. The staining distribution was classified as follows: < 25% (0.1), 26%–50% (0.6), 51%–75% (0.9), and 76%–100% (1.0). The staining intensity was rated as follows: 0 (absent), 0.5 (minimal), 1 (slight), 2 (moderate), and 3 (intense). The immunohistochemical staining score was determined by multiplying the distribution score by the intensity score [17].

### 2.4 Assessment of Serum SQSTM1 (p62) Levels

Serum SQSTM1 (Sequestosome‐1) levels were determined using a commercially available sandwich ELISA kit (Rat SQSTM1 ELISA Kit, Cat. No: ER7180, FineTest, Wuhan, China), according to the manufacturer's instructions. Absorbance was measured at 450 nm, and concentrations were calculated using a four‐parameter logistic (4‐PL) calibration curve. Results were expressed as ng/mL.

### 2.5 Tissue Preparation for Biochemical Analyzes

As stated in our previous study [2], we weighed the left kidney tissues of each rat and washed them with phosphate‐buffered saline (PBS) at pH 7.4. The tissues were subsequently homogenized in precooled PBS, and the resultant kidney homogenates were gathered as suspensions. We measured the protein concentration in the left kidney tissues use the Bradford protein assay kit (Bio‐Rad, 5000201).

### 2.6 Assessment of Kidney Function and Selected Blood Markers

We permitted the rat blood samples to coagulate at ambient temperature for 30 min, thereafter, subjecting them to centrifugation at 1500*g* for 10 min at +4°C. The resultant supernatant was meticulously transferred to new microcentrifuge tubes. Subsequently, we assessed some blood markers, including blood urea nitrogen (BUN) and creatinine (CREA), Uric acid (UA), lactate dehydrogenase (LDH), Albumin (ALB) and Total protein (TP) employing established laboratory protocols.

### 2.7 Assessment of Oxidative Stress‐Related Markers

Malondialdehyde (MDA), a key marker of oxidative stress in cells and tissues, was quantified using the method previously outlined [18]. The protein concentrations of each kidney tissue sample were determined. Following incubation, absorbance readings were measured via a microplate reader (Thermo Fisher Multiscan Go).

### 2.8 Assessment of Antioxidant Capacity

Serum superoxide dismutase (SOD) and glutathione peroxidase (GPx) levels were measured using commercially available ELISA kits according to the manufacturer's instructions (BT‐LAB; Cat. Nos. E2269Ra and E1242Ra, respectively). Using a colorimetric kit (Elabscience, E‐BC‐K801‐M), we determined the serum total antioxidant status (TAS). The results were reported as mmol Trolox equivalents per litre (mmol Trolox Eq./L). A different colorimetric kit (Elabscience, E‐BC‐K802‐M) was used to measure the serum total oxidant status (TOS). The results were expressed as micromoles of hydrogen peroxide equivalents per litre (µmol H_2_O_2_ Eq./L). The total oxidant status (TOS) was divided by the total antioxidant status (TAS) to determine the oxidative stress index (OSI), as previously explained [19]. The following formula was used to represent the OSI in arbitrary units: OSI (arbitrary unit) = [TOS (µmol H_2_O_2_ Eq./L)/TAS (µmol Trolox Eq./L)] × 100.

### 2.9 Statistical Analyzes

We analyzed all statistical data using GraphPad Prism (version 6.0). We utilized the Shapiro–Wilk test to evaluate data distribution. Subsequently, we utilized the *Kruskal–Wallis H* test for group comparison, followed by the *Mann–Whitney U* test for pairwise analyzes. We reported the results as mean ± standard deviation and deemed *p*‐values < 0.05 as statistically significant.

## 3 Results

### 3.1 Sinapic Acid Attenuates LPS‐Induced Tubular Damage and Serum Biomarker Dysregulation

**Figure 2:**
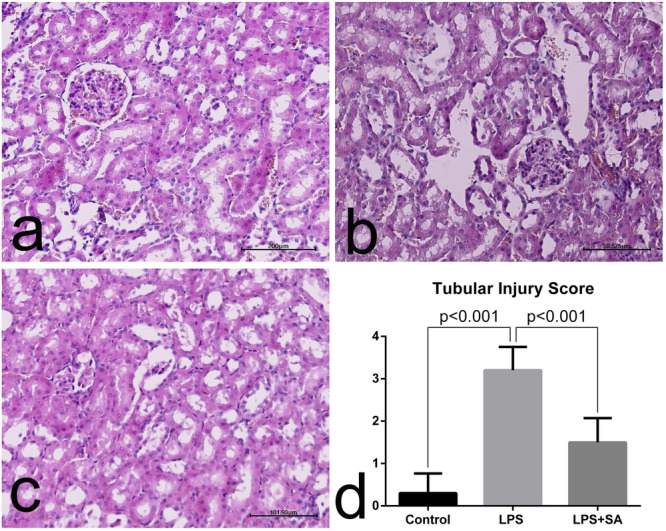
Effects of SA on renal histopathology and tubular injury scores in LPS‐induced AKI rats. Representative hematoxylin and eosin (H&E)‐stained kidney sections from (a) control, (b) LPS, and (c) LPS + SA groups. The control group shows normal renal architecture, whereas the LPS group exhibits marked tubular epithelial cell injury, luminal dilatation, and loss of brush border integrity. SA pretreatment attenuated these histopathological alterations. (d) Quantitative tubular injury scores reveal a significant increase in injury in the LPS group compared to control, with SA pretreatment significantly reducing the severity of damage (*p* < 0.001 vs. LPS). Statistical analysis was performed using the *Kruskal–Wallis H* test followed by the *Mann–Whitney* U test. *p* values are indicated in the graphs. Data are presented as mean ± SD (*n* = 8 per group). LPS, lipopolysaccharide; SA, sinapic acid.

**Figure 3:**
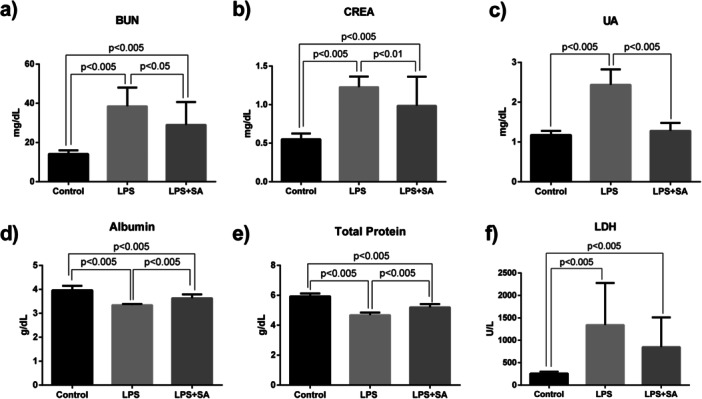
Effects of SA on serum biochemical parameters in LPS‐induced AKI in rats. Serum levels of (a) BUN, (b) CREA, (c) UA, (d) albumin, (e) total protein, and (f) LDH were measured in control, LPS, and LPS + SA groups. Data are presented as mean ± SD (*n* = 8 per group). Statistical analysis was performed using the *Kruskal–Wallis H* test followed by the *Mann–Whitney* U test. *p* values are indicated in the graphs. *p* values are indicated in the graphs. SA pretreatment (40 mg/kg/day, orally for 7 days) significantly ameliorated LPS (5 mg/kg)‐induced alterations in renal function and other markers, indicating its nephroprotective potential. BUN, blood urea nitrogen; CREA, creatinine; LDH, lactate dehydrogenase; LPS, lipopolysaccharide; SA, Sinapic acid; UA, uric acid.

We examined the potential therapeutic impact of SA on renal tubular damage in an LPS‐induced AKI model and depicted the histological alterations in H&E‐stained kidney sections, together with tubular injury scores, in Figure 2. The renal tissue in the control group displayed a normal histological structure (Figure 2a). Conversely, the LPS group exhibited significant damage confined to the epithelial cells of both the proximal and distal tubules (Figure 2b). The pre‐treatment with SA substantially alleviated the tubular injury induced by LPS as illustrated in Figure 2d. The statistical evaluation revealed a significant increase in tubular damage scores in the LPS group when compared to the control group (Figure 2d, *p* < 0.001). We further investigated the BUN, CREA and UA concentrations in a rat model of sepsis‐induced AKI to assess the effects of SA and LPS on renal function, with the findings illustrated in Figure 3. Following the administration of LPS, serum BUN, CREA and UA concentrations exhibited a statistically significant increase compared to the control group (Figure 3a,b,c) (*p* < 0.005). The elevations in BUN, CREA and UA induced by LPS in the AKI rat model were mitigated by SA treatment (Figure 3a,b,c) (*p* < 0.05). We additionally conducted an analysis of serum albumin, total protein and LDH levels in order to assess the impact of SA and LPS on serum biomarkers, as illustrated in Figure 3. The administration of LPS resulted in a significant reduction in both albumin and total protein levels, whereas treatment with SA attenuated the decreases induced by LPS (Figure 3d,e, *p* < 0.005). Our data revealed that SA pretreatment significantly attenuated the LPS‐induced elevations in serum LDH levels, as shown in Figure 3.

#### 3.1.1 Sinapic Acid Attenuates LPS‐Induced Changes in Autophagy‐Related, Apoptotic, and Inflammatory Markers

**Figure 4:**
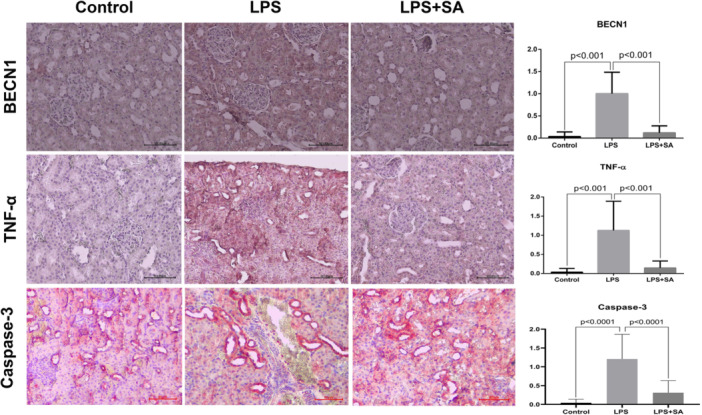
Effects of SA on BECN1, TNF‐α, and Caspase‐3 expression in renal tissues of LPS‐induced AKI rats. Representative immunohistochemical staining images show BECN1 (autophagy‐related marker), TNF‐α (inflammatory marker), and Caspase‐3 (apoptosis‐associated marker) immunoreactivity in kidney sections from the control, LPS, and LPS + SA groups (scale bar = 100 μm). LPS administration markedly increased BECN1, TNF‐α, and Caspase‐3 expression compared to the control group, whereas SA pretreatment attenuated these elevations (*p* < 0.001 for BECN1 and TNF‐α; *p* < 0.0001 for Caspase‐3, vs. LPS). Statistical analysis was performed using the Kruskal–Wallis H test followed by the Mann–Whitney U test. Data are presented as mean ± SD (*n* = 8 per group). BECN1, beclin‐1; LPS, lipopolysaccharide; SA, sinapic acid; TNF‐α, tumor necrosis factor‐alpha.

**Figure 5:**
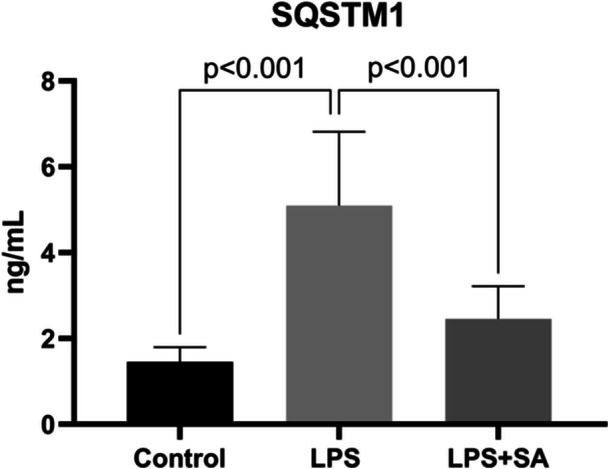
Effects of SA on circulating SQSTM1/p62 levels in rats with LPS‐induced AKI. LPS administration significantly increased serum SQSTM1 levels compared with the control group (*p* < 0.001), whereas SA pretreatment significantly attenuated this increase (*p* < 0.001). Serum SQSTM1 was evaluated as an autophagy‐related marker and not as a direct measure of renal autophagic flux. Data are presented as mean ± SD (*n* = 8 per group). Statistical analysis was performed using the Kruskal–Wallis H test followed by the Mann–Whitney U test. LPS, lipopolysaccharide; SA, Sinapic acid; SQSTM1, sequestosome 1.

We additionally evaluated renal BECN1, TNF‐α, and Caspase‐3 immunoreactivity to examine the effects of LPS and SA pretreatment on autophagy‐related, inflammatory, and apoptosis‐associated markers, respectively, in the LPS‐induced AKI model (Figure 4). Pre‐treatment with SA significantly attenuated the LPS‐induced elevation of BECN1, an essential mediator of autophagy, in renal tissues (Figure 4a, *p* < 0.001). Moreover, we evaluated the expression levels of TNF‐α, a pivotal pro‐inflammatory mediator, in renal tissue. The administration of LPS resulted in a substantial increase in TNF‐α expression (Figure 4b, *p* < 0.001), whereas SA pre‐treatment markedly mitigated these elevations (Figure 4b, *p* < 0.001). We further assessed Caspase‐3 expression in renal tissue to determine whether LPS‐induced injury was accompanied by activation of the apoptotic cascade (Figure 4c). LPS administration markedly increased Caspase‐3 expression compared to the control group (*p* < 0.0001), consistent with tubular epithelial apoptosis. SA pretreatment significantly reduced LPS‐induced Caspase‐3 expression (*p* < 0.0001), with values approaching those observed in the control group, indicating a robust anti‐apoptotic effect of SA pretreatment. To provide complementary evidence beyond Beclin‐1 expression, we measured serum SQSTM1/p62 levels (Figure 5). LPS administration significantly increased serum SQSTM1 levels compared to the control group (*p* < 0.001), indicating alterations in this autophagy‐related substrate alongside the concurrent rise in BECN1 expression. SA pretreatment significantly attenuated the LPS‐induced increase in serum SQSTM1 levels (*p* < 0.001), indicating that its protective effects were accompanied by changes in the evaluated autophagy‐related markers.

### 3.2 Effects of SA on Oxidative Stress Parameters in the Context of LPS‐Induced AKI

**Figure 6:**
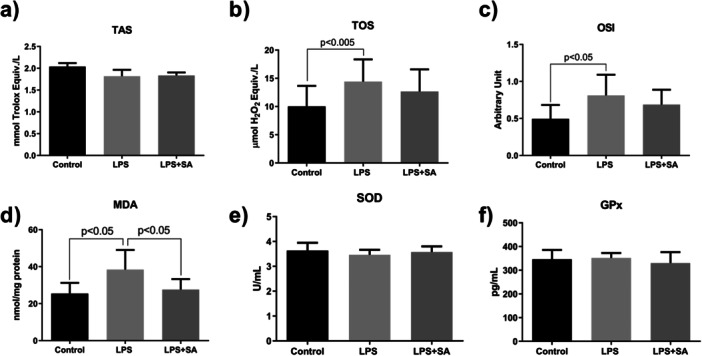
Effects of SA on oxidative stress parameters in LPS‐induced AKI in rats. Serum TAS (a), TOS (b), OSI (c), SOD (e), and GPx (f) levels and renal MDA (d) levels were evaluated in the control, LPS, and LPS + SA groups. Data are presented as mean ± SD (*n* = 8 per group). Statistical analysis was performed using the Kruskal–Wallis H test followed by the Mann–Whitney U test. *p* values are indicated in the graphs. LPS administration significantly increased TOS, OSI, and renal MDA levels compared with the control group. SA pretreatment significantly attenuated the LPS‐induced increase in renal MDA but did not significantly alter SOD, GPx, or TAS. These findings indicate a selective reduction in lipid peroxidation rather than a generalized enhancement of antioxidant defenses. GPx, glutathione peroxidase; LPS, lipopolysaccharide; MDA, malondialdehyde; OSI, oxidative stress index; SA, Sinapic acid; SOD, superoxide dismutase; TAS, total antioxidant status; TOS, total oxidant status.

We meticulously evaluated the effects of SA on the oxidative stress levels present in the renal tissues and serum samples of a rat model that was subjected to AKI via LPS administration, and the resultant data have been comprehensively illustrated in Figure 6. The administration of LPS led to a statistically significant elevation in total oxidative status (TOS) as depicted in Figure 6b (*p* < 0.005), alongside a notable increase in oxidative stress index (OSI) as shown in Figure 6c (*p* < 0.05), and malondialdehyde (MDA) levels as represented in Figure 6d (*p* < 0.05) when compared to the control group, thereby indicating a pronounced enhancement in oxidative stress. In contrast, the pretreatment with SA effectively mitigated the rise in MDA levels that was induced by the administration of LPS, as evidenced in Figure 6d (*p* < 0.05). However, it is important to note that no statistically significant differences were detected among the various experimental groups concerning TAS as illustrated in Figure 6a, SOD levels depicted in Figure 6e, or GPx levels represented in Figure 6f. Collectively, these findings demonstrate that SA pretreatment attenuated the LPS‐induced increase in renal MDA levels without significantly altering serum SOD, GPx, or TAS levels.

## 4 Discussion

The inflammatory response induced by sepsis can lead to multi‐organ failure, particularly acute kidney injury (AKI), and significantly contributes to elevated morbidity and mortality rates [20]. Despite significant advancements in medical research and technological innovations, there remains an absence of efficacious treatment modalities for AKI at present. Thus, it is imperative to investigate novel pharmacological agents in order to improve the treatment strategies for AKI. SA, possessing significant antioxidant and anti‐inflammatory characteristics, is a promising therapeutic candidate; nevertheless, its prospective effectiveness in LPS‐induced AKI remains ambiguous [10]. Therefore, we examined whether SA pretreatment attenuates LPS‐induced renal injury and dysfunction and whether these effects are accompanied by changes in autophagy‐related, apoptotic, inflammatory, and oxidative stress markers. LPS is extensively employed to establish animal models of AKI, and its intraperitoneal administration results in elevated renal injury biomarkers and renal damage within a timeframe of 4 h and thereafter [2, 21]. Our histopathological analyzes demonstrated that the administration of LPS caused substantial structural damage to the renal tubules, supporting previous research that identifies epithelial cell injury as a principal characteristic of sepsis‐related AKI [2]. The significant rise in tubular damage scores seen in the LPS‐treated group confirms the severity of injury in both proximal and distal tubules. Pretreatment with SA markedly alleviated these pathological alterations, indicating its potential effectiveness as a renoprotective drug in cases of endotoxin‐induced renal injury. The substantial elevations in serum levels of BUN, CREA, and UA following LPS administration, as recorded in our study, indicate severe renal dysfunction, supporting findings from previous research using experimental models of sepsis‐induced AKI. These biomarkers are recognized indicators of reduced glomerular filtration rate and tubular dysfunction, with elevated levels signifying both impaired clearance and structural damage of the nephron [22].

The current findings demonstrate alterations in autophagy‐related markers in LPS‐induced AKI, as indicated by increased renal BECN1 immunoreactivity and circulating SQSTM1 levels following LPS administration. These findings are consistent with previous studies suggesting that autophagy‐related responses may be involved in both protective and deleterious processes during septic renal injury [3, 23]. Similar observations have been reported in other LPS‐induced inflammatory models. For example, in human dental pulp cells, LPS stimulation increased the expression of autophagy markers BECN1 and LC3‐II [24]. Autophagy is widely acknowledged as a cytoprotective mechanism that sustains cellular homeostasis and promotes the elimination of damaged organelles and proteins [3, 24]. In the context of acute inflammatory stress, excessive or dysregulated autophagy may shift from a beneficial adaptive response to a harmful maladaptive one, leading to the death of tubular epithelial cells and impairing regenerative capacity [25]. This dichotomy has been well recorded in the context of sepsis‐related kidney damage, highlighting the essential importance of sustaining a balanced autophagic response. Moreover, SA pretreatment significantly reduced renal BECN1 immunoreactivity, indicating attenuation of the LPS‐induced change in this autophagy‐associated marker. Importantly, Beclin‐1 alone reflects autophagy initiation rather than completed autophagic flux [5]. To provide complementary evidence, we additionally assessed serum SQSTM1/p62, an autophagy‐related substrate that is normally degraded following successful autophagosome–lysosome fusion. LPS administration markedly increased circulating SQSTM1 levels alongside elevated renal BECN1 immunoreactivity. This concurrent pattern may be consistent with altered autophagic processing and possible impairment of downstream clearance; however, it does not establish blocked autophagic flux because SQSTM1 was measured in serum at a single time point. SA pretreatment reduced both renal BECN1 immunoreactivity and circulating SQSTM1 levels, suggesting that its protective effects are accompanied by attenuation of LPS‐induced alterations in autophagy‐related markers. Nevertheless, renal LC3‐I/LC3‐II analysis, tissue SQSTM1/p62 assessment, and dynamic autophagic flux experiments are required to determine whether and how SA influences renal autophagic processing.

Similar benefits have been recorded for several polyphenolic substances, where their antioxidant properties and regulatory impact on signaling pathways have been linked to improved renal outcomes [25]. Furthermore, contradictory evidence from colitis and intestinal fibrosis models indicates that SA can enhance Beclin1 expression and stimulate autophagy [26]. These discrepancies may arise from variances in disease models, damage severity, and the timing of autophagy activation. Sustained autophagy may facilitate extracellular matrix destruction and mitigate fibrosis in intestinal fibrosis; however, excessive activation during acute inflammation in sepsis‐induced AKI could exacerbate tubular injury. Variations in SA dosage, treatment duration, and delivery timing may elucidate these disparate findings. These context‐dependent effects may be relevant to sepsis‐induced AKI; however, functional autophagic flux studies are required to determine whether SA directly influences autophagic processing in this model. However, differing perspectives in the academic literature indicate that the activation of autophagy can play either a protective or detrimental role depending on the timing, intensity, and contextual factors of the injury, underscoring the need for mechanistic studies to clarify optimal modulation strategies.

Alongside the observed changes in autophagy‐related markers, our data demonstrate significantly increased renal TNF‐α immunoreactivity following LPS administration. TNF‐α serves as a pivotal mediator in the pathophysiology of sepsis‐related renal impairment, promoting the recruitment of leukocytes, exacerbating oxidative stress, and initiating pro‐apoptotic signaling cascades that culminate in tubular injury [27]. This finding is consistent with prior reports describing anti‐inflammatory effects of SA in other injury models, in which these effects have been associated with inhibition of the NF‐κB signaling pathway and downstream cytokine downregulation; however, NF‐κB signaling and its downstream cytokines were not directly assessed in the present study, and this comparison should therefore be regarded as contextual rather than mechanistic evidence from our own data [28, 29]. The concurrent attenuation of LPS‐induced changes in autophagy‐related markers and renal TNF‐α immunoreactivity suggests that the protective effects of SA may be associated with multiple biological processes; however, additional inflammatory and mechanistic analyzes are required to confirm this interpretation.

In addition to its associations with autophagy‐related and inflammatory markers, SA pretreatment markedly attenuated LPS‐induced Caspase‐3 immunoreactivity in renal tissue, indicating a reduction in this apoptosis‐associated marker. Apoptosis represents a convergent downstream consequence of the inflammatory and oxidative insults triggered by LPS; TNF‐α signaling and excessive ROS generation are both well‐established activators of the intrinsic and extrinsic apoptotic pathways, culminating in Caspase‐3‐mediated execution of cell death [9]. The reduction in renal Caspase‐3 immunoreactivity following SA pretreatment suggests that modulation of apoptosis‐associated processes may contribute to the renoprotective effects of SA. The reduction in Caspase‐3 immunoreactivity occurred alongside attenuation of tubular injury in SA‐pretreated animals. Although these concurrent findings suggest a possible relationship between apoptosis‐associated changes and renal protection, additional mechanistic studies are required to establish causal links among the evaluated pathways. Oxidative stress represents a fundamental pathogenic mechanism underlying sepsis‐ AKI, playing a pivotal role in both the structural and functional impairment of renal tissue [30]. Our data indicated that the treatment of LPS caused a substantial increase in TOS, OSI, and MDA levels, consequently indicating an enhancement in lipid peroxidation and a shift towards a pro‐oxidant environment. These observations align with earlier studies indicating that LPS induces a heightened production of ROS, which surpasses the capacity of endogenous antioxidant defences and aggravates tubular damage [2]. SA pretreatment specifically reduced the LPS‐induced increase in MDA levels, suggesting that its primary antioxidant effect in this experimental context is the suppression of lipid peroxidation, consistent with previous studies [28]. The reduction of MDA, a persistent consequence of ROS‐induced damage to membrane lipids, indicates enhanced preservation of cellular integrity in renal tissues. Nonetheless, the lack of significant differences in TAS, SOD, and GPx levels between groups suggests that the protective effect of SA may not involve a broad enhancement of overall antioxidant capacity, but rather a targeted attenuation of specific oxidative damage pathways. This apparent lack of effect on general antioxidant parameters may be attributable to the SA dosage and duration of administration used in the present study. This dissociation between lipid peroxidation and enzymatic antioxidant activity has been reported in other acute LPS‐induced injury models and may reflect differing kinetics: MDA accumulation reflects an immediate consequence of ongoing membrane lipid peroxidation, whereas compensatory changes in antioxidant enzyme expression or activity (SOD, GPx) typically require longer exposure or transcriptional upregulation, which may not be fully captured within the 6‐h post‐LPS window used in this acute model. Additionally, TAS reflects the aggregate, non‐enzymatic antioxidant capacity of serum and may be less sensitive to localized renal tissue redox changes than tissue‐specific lipid peroxidation markers. Taken together, these findings suggest that at this early time point, SA's protective effect is more readily detected as attenuation of lipid peroxidation than as measurable enhancement of enzymatic antioxidant defenses, and that longer or repeated‐dosing protocols may be needed to reveal effects on SOD, GPx, and TAS. All in all, these data indicate that SA's antioxidant activity in LPS‐induced AKI is predominantly directed at lipid peroxidation, alongside its associations with reduced inflammatory and autophagy‐related marker levels. A combined assessment of oxidative stress, inflammation, and autophagy‐related markers may offer a more holistic view of the therapeutic potential of SA in alleviating renal impairment in sepsis. While our study provides novel insights into the associations between SA pretreatment and autophagy‐related, apoptotic, inflammatory, and oxidative stress markers in LPS‐induced AKI, several limitations must be acknowledged. First, our assessment of autophagy‐related changes was based on renal BECN1 immunoreactivity and circulating SQSTM1/p62 levels. Renal LC3‐I/LC3‐II conversion, tissue‐level SQSTM1/p62 assessment, and dynamic autophagic flux assays were not performed. Therefore, these findings should be interpreted as alterations in autophagy‐related markers rather than evidence of impaired or restored renal autophagic flux. Second, our evaluation of the inflammatory response was limited to renal TNF‐α immunoreactivity; other key mediators such as IL‐6 and IL‐1β, as well as upstream signaling pathways including NF‐κB activation and inflammatory‐cell infiltration, were not assessed, leaving the anti‐inflammatory mechanism of SA incompletely characterized. Third, the experimental design did not include an SA‐only (Control + SA) group; therefore, we cannot determine whether SA independently affects normal renal structure or baseline biochemical, autophagy‐related, apoptotic, inflammatory, and oxidative stress‐related parameters. Fourth, this study exclusively employed male Wistar albino rats, neglecting any sex‐related differences in immunological modulation, oxidative stress responses, and susceptibility to sepsis‐induced kidney damage. This approach successfully minimized hormonal variability but limits the generalizability of our findings to female populations; including both sexes in future studies will be essential to clarify potential sex‐specific responses to SA therapy. Fifth, we administered a single, moderate dose of LPS (5 mg/kg) to induce AKI while reducing the risk of severe systemic failure; the use of varied dosing regimens or alternative injury models may provide a more comprehensive understanding of the therapeutic window for sepsis‐related AKI. Finally, our study was limited to a pretreatment framework, which does not establish whether SA administration would be effective after the onset of AKI. Future studies incorporating post‐treatment protocols, a dedicated SA‐only control group, dose‐response designs, direct measurement of IL‐6, IL‐1β, and NF‐κB signaling, and functional autophagic flux assays are needed to comprehensively characterize the therapeutic potential and mechanistic basis of SA in sepsis‐related AKI.

## 5 Conclusion

Our data presented here, for the first time, show that SA attenuates sepsis‐induced AKI, an effect accompanied by attenuation of LPS‐induced alterations in autophagy‐related markers (BECN1, SQSTM1), suppression of apoptotic cell death (Caspase‐3), attenuation of TNF‐α–mediated inflammation, and reduction of lipid peroxidation. In this model, LPS administration increased renal BECN1 and TNF‐α immunoreactivity and renal MDA levels, whereas SA pretreatment attenuated these changes, reduced tubular injury, and improved serum markers of renal dysfunction. These findings support the nephroprotective potential of SA pretreatment in experimental LPS‐induced AKI. However, the underlying mechanisms and clinical relevance of these effects require further investigation. Future studies incorporating post‐LPS treatment protocols, different SA doses, animals of both sexes, and mechanistic approaches such as pathway inhibition or gene knockdown are needed to clarify the therapeutic efficacy, underlying mechanisms, and translational relevance of SA in LPS‐induced AKI.

## Author Contributions

**Serdar Doğan:** conceptualization, methodology, formal analysis, investigation, resources, supervision, writing – review and editing, writing – original draft, funding acquisition, project administration. **Hamza Malik Okuyan:** conceptualization, methodology, formal analysis, project administration, resources, supervision, writing – original draft, writing – review and editing, funding acquisition. **Ayça Coşkun:** conceptualization, investigation, methodology, formal analysis, writing – review and editing, writing – original draft. **Şeyda Öznur Ayçiçek Özen:** writing—original draft, writing – review and editing, methodology, investigation. **Mehmet Doğan:** writing – original draft, writing – review and editing, methodology, investigation. **İhsan Karaboğa:** investigation, methodology, writing – review and editing, writing – original draft, resources, supervision. **Sibel Kulaksızoğlu:** investigation, methodology, writing – original draft, writing – review and editing, resources, supervision.

## Ethics Statement

The present study was conducted in compliance with the Institutional Animal Care and Use Committee Guidelines, and all experimental protocols were approved by Sakarya University's local Animal Ethics Committee (Approval number: 2023‐14).

## Conflicts of Interest

The authors declare no conflicts of interest.

## Data Availability

The data that support the findings of this study are available from the corresponding author upon reasonable request.
